# Parent-adolescent sexual and reproductive health information communication in Ghana

**DOI:** 10.1186/s12978-025-01961-y

**Published:** 2025-02-19

**Authors:** Frank Bediako Agyei, Doreen K. Kaura, Janet D. Bell

**Affiliations:** Department of Nursing and Midwifery, Faculty of Medicine and Health Sciences, Stellenbosch University, Cape Town, South Africa

**Keywords:** Parent, Adolescent, Sexual and Reproductive Health, Information, Communication, Intervention, Lower- and middle-income countries, Systematic review

## Abstract

**Background:**

A culturally sensitive sexual and reproductive health (SRH) information communication intervention which is effective can improve SRH information communication (IC) between parents and their adolescents. This facilitates adolescents’ informed SRH decisions to optimise positive SRH outcomes.

**Aim:**

The aim of this article was to integrate the findings from a systematic review and a qualitative study on sexual and reproductive health information communication and the considerations to make in adapting an effective parent-adolescent SRH information communication intervention from the systematic review findings.

**Methods:**

Explanatory sequential Mixed Methods Research was used; first, a quantitative Systematic Review was conducted in lower-and-middle-income countries, utilising Joanna Briggs Institute (JBI) software for reviews. The systematic review findings were then explained, utilizing an exploratory qualitative design in the second phase of the study. A purposive sample of ten parent-adolescent pairs was selected from Asante Akyem North Municipality of Ghana and all participants were interviewed individually. The sample was based on the demographics highlighted in the systematic review. A semi-structured interview guide was developed from the findings of the systematic review.

**Results:**

The results confirmed that effective SRH information communication interventions are associated with parent-adolescent SRH information communication skills. SRH communication is also influenced by the SRH information parents and adolescents have and the personal and social motivation to communicate the information. The method of intervention delivery, the experts involved, and the place of delivery were also identified as important issues to consider in adapting and implementing an intervention.

**Conclusion:**

The study has provided information on the components of a culturally sensitive SRH information communication intervention. The contextual information gathered, which explained the systematic review findings, will be helpful in the adaptation of SRH information communication intervention.

## Introduction

Adolescent Sexual and Reproductive Health (SRH) has received attention globally because of the consequences of risky adolescent sexual behaviour, such as early sexual debut, unprotected sex, and multiple sexual partners [1]. Such behaviours lead to sexually transmitted infections (STIs) including HIV/AIDS, unplanned pregnancies with their associated consequences such as unsafe abortion, poor birth outcomes and school dropout [2].

In lower- and middle-income countries (LMICs), adolescents have challenges in meeting their SRH needs, and risky sexual behaviours are more pronounced in such countries [3]. Adolescents face barriers in assessing SRH information from reliable sources such as parents, who are their preferred source of received information.

Approximately 13% of adolescent girls and young women globally give birth before age 18 [4]. Sub-Saharan Africa has the highest adolescent birth rates globally, with 93 births per 1,000 girls aged 15–19 years [5]. In Ghana, the pooled prevalence of adolescent pregnancy is approximately 15.4%, with higher rates in rural areas (19.5%) compared to urban areas (10.6%) [6].

In Ghana, there is an increased rate of adolescent pregnancies [7]. This may be because adolescents lack SRH education, are neglected by their parents and face sexual abuse [8]. In curbing these issues, parent-adolescent SRH information communication has been cited to be one of the most important activities [9]. Through this, adolescents gain information that helps them with sexual decision-making, which contributes to improved SRH outcomes [10]. The ability of adolescents to make decisions based on accurate SRH information is very important. SRH information needs to be not only accessible, but also comprehensible to the adolescent [11]. Research has shown that when adolescents acquire knowledge from SRH information communication, they may avoid or delay sexual activities and establish healthy sexual activity in the future [9]. In phase one of this study, which was the systematic review, SRH information identified included (but was not limited to) changes during adolescence, menstruation, sex, abstinence, dating, relationships, pregnancy, safer sex, contraceptives, STIs including HIV/AIDS, contraceptives, abortion, and sexual coercion resistance [1, 9, 13–15]. Adolescents’ acceptance of SRH information also depends on its source. In the second phase of this study, adolescents mentioned that they prefer receiving SRH information from their parents. This is also in line with the findings of other studies [16]. Parents play an influential role in educating adolescents on SRH; therefore, it is important to look at parent-adolescent SRH information communication.

Parent-adolescent SRH information communication is how parents share information, influenced by their values, beliefs, and standards regarding SRH with adolescents. This could influence the knowledge, attitudes and behaviour of adolescents regarding SRH [13]. SRH information communication between parents and adolescents is influenced by the communication skills possessed by both parents and adolescents [14]. Parents as well as adolescents must have some communication skills to be able to share SRH information. This skill needs may be met when parents and adolescents have knowledge and skill to communicate SRH information [9] using a SRH information communication intervention that is culturally sensitive.

A culturally sensitive SRH information communication intervention is one that intentionally takes into consideration the parent and adolescent’s values, beliefs and standards to increase the SRH communication skills of both parents and adolescents [17]. Such an intervention is likely to be accepted by people in the Ghanaian context since it takes into account their cultural predisposition.

Considering the above, it is important to integrate the findings from the systematic review and the qualitative studies to inform the adaptation or adoption of a culturally sensitive SRH information communication intervention in Ghana.

The information, motivation, behavioural skill model [18] was used to organize the findings from the systematic review and the qualitative studies for analysis to be made.

## Research design and methods

### Research design

This is the fourth of a series of articles on an explanatory sequential mixed methods study on parent-adolescent SRH information communication intervention in Ghana. The first phase was a systematic review of effective interventions for SRH information communication utilising JBI SUMARI and the Preferred Reporting Items for Systematic Reviews and Meta-Analyses (PRISMA). Further, an exploratory design was used to explain the findings from the systematic review. The integration of findings was elucidated through merging, connecting and building of meta inferences. The connecting scheme for data integration as proposed by [19] was used because data from the qualitative phase was used to explain the systematic review results. The mixed data analysis proposed by Onwuegbuzie and Teddlie [20] was used to combine the data.

**Fig. 1:**
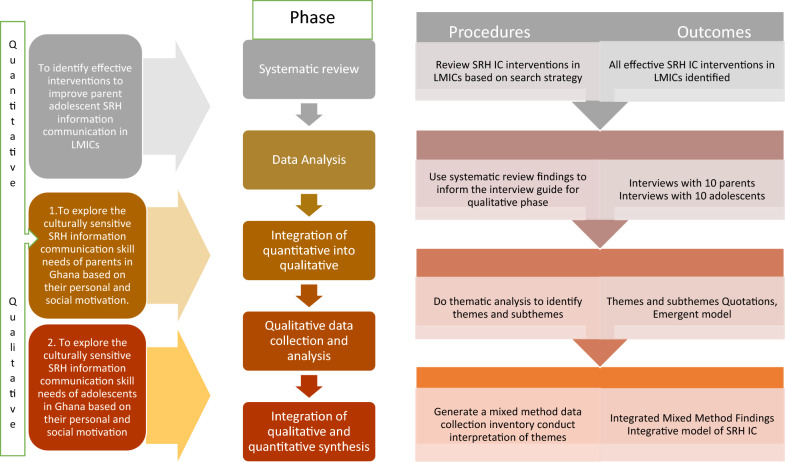
The mixed method design

First, integrated data reduction was conducted. The statistics from the individual studies identified from the systematic review were computed together with the themes generated from the qualitative studies. The researcher then did a parallel graphical representation of quantitative and qualitative data (tables for quantitative parameters and thematic matrices for qualitative parameters) to visually compare the data. Cross-case analysis was done to ensure quantification and qualification. Qualitative and quantitative data were then correlated. Multiple data sets were then merged to create new codes and variables. Finally, meta inferences were derived by reviewing data from both quantitative and qualitative data sets. The steps taken has been summarised on Fig. 1 below.

### Study setting

The study began with a systematic review of sexual and reproductive health (SRH) information communication intervention in low- and middle-income countries (LMICs). Following this, a qualitative descriptive phase was conducted in the Asante Akyem North Municipality of Ghana, an LMIC, to provide localized insights into SRH communication practices. Asante Akim North municipality has a large population of adolescents which is diverse in culture and religion. Such a setting provides an opportunity to gather in-depth data to accurately adapt an intervention for parent-adolescent information communication on SRH that is context specific.

The municipality is located in the eastern part of the Asanti region. About 98.2% of the population in the municipality are Ghanaians (95.9% by birth, 0.6% by naturalization and 1.7% with dual nationality) and 0.9% are from other Economic Community of West African States (ECOWAS) countries. With regards to religion, the majority (83.7%) of the persons in the district are Christians, 9% are Muslims and 5.6% do not associate with any religion. This is important to note because religion has been linked with communication and decision making in health [21]. The main languages for communication in the municipality are English and ‘Twi’ languages. Other languages spoken are based on ethnicity. Language is not just considered as part of culture but helps in the transmission of culture [22]. The majority (79.2%) are literate. There are 16,038 adolescents in the district [23].

### Description of quantitative phase

The quantitative phase was a systematic review of quantitative studies, which was guided by the preferred reporting items for systematic reviews and meta-analyses (PRISMA) and the Joana Briggs Institute (JBI) manual. The review was registered in PROSPERO with registration number, CRD42022297526.

*Information Sources and Search Strategy* a search strategy to search for studies published from January 2011 to December 2021 in EMBASE, CINAHL, PubMed, OVID, Scopus, Cochrane Reviews Library, Web of Science and Science Direct. The keywords used for the search included (Adolescents” OR “Teenagers” OR “Young Adults”) AND (“Parents” OR “Caregiver” OR “Mother” OR “Father” OR “Guardian”) AND (“Sexual” AND “Reproductive” AND “Health”) AND (“Information” AND “Communication”) AND (“Interventions” OR “Strategies” OR “Best Practices”). Studies that trained parents, adolescents (13 to 16 years of age) or both on SRH information communication was selected. The authors considered both experimental and quasi-experimental designs, including randomized control trials, in LMICs.

*Study Selection* After the search in the above-mentioned databases, the citations identified were uploaded into Mendeley (1.19.8) and screened by two reviewers. Potentially relevant studies were imported into the JBI system for the unified management, assessment, and review of information (JBI SUMARI). The inclusion criteria were used to assess the full text of the studies by the researcher and his supervisor, and the results were reported.

*Inclusion and Exclusion Criteria* this review included both experimental and quasi-experimental study designs, including randomized controlled trials in LMICs, that exposed parents, adolescents (13 to 16 years) or both to SRH communication interventions. This age group comprises adolescents in the latter stage of early adolescence and those in middle adolescence and represents a transition from early to late adolescence. Since some studies did not specifically include this age group, relevant studies were included if at least 50% of the participants were between the ages of 13 and 16; results were stratified according to age groups.

*Assessment of methodological quality / critical appraisal* included studies were critically appraised by the researcher and his supervisor independently, using the JBI standardized critical appraisal tools [24].

*Data Extraction and Synthesis* Standardized data such as authors, study aims, participants, and settings were extracted from each study, as well as information regarding the intervention components and outcomes. A narrative synthesis approach was used to synthesize data because of the heterogenous nature of the study outcomes and the intervention approaches.

### Description of the qualitative phase

*Study Population and Sampling* In the qualitative phase, Parent adolescent dyads were recruited but interviewed separately. Parents included a biological father or mother, or the male or female guardians of an adolescent (13 to 16 years of age) who were willing to participate in the study. Adolescents aged 13 to 16 years were included in the study because the purposive sampling approach was used to sample parents and adolescents. Flyers with information were distributed to potential participants. Parental consent and child assent forms were signed by parents and adolescents respectively prior to inclusion in the study. To include the nuances of the developmental needs of adolescents, the authors included 5 parents of adolescents aged 13 to 14 years and 5 parents whose adolescents were 15 to 16 years of age. Maximum variability was ensured by sampling at least three male and three female parents. Adolescents who were cohabiting or married were excluded from the study, as were their parents. The final sample comprised 10 parents and 10 adolescents based on saturation. Data saturation was achieved after the 8th parent-adolescent pair, as no new themes or codes emerged during the iterative process of data analysis. To validate this, two additional interviews were conducted, which further confirmed the recurring themes. This approach aligns with established qualitative research guidelines that define saturation as the point at which additional data does not contribute new information or insights [25]

*Data Collection* In the qualitative phase, individual interviews were conducted, using a semi-structured interview guide. The interview guide was developed by the researcher in consultation with the supervisory team, based on the findings of the systematic review and the IMB skills model, to obtain data from parents and adolescents. The guide comprised open-ended questions with probes and prompts to ensure that spontaneous, rich and vivid data was obtained from the context.

A pilot interview was initially conducted with a parent and her adolescent separately to assess the clarity of the guide and the time estimated for the interviews. The data was transcribed and analysed and afterwards discussed with the supervisors. The interview guide was slightly modified after the pilot study, to ensure that the SRH information communication skill needs would be well explored.

Consent was obtained from parents and child assent was obtained from adolescents. After that, interviews were conducted at a place and time convenient to the participants. The researcher scheduled a convenient date, time and venue, suitable for participants. Since parents preferred interviews to be conducted in their home environments, both parent and adolescent interviews took place in the same environment, although they were interviewed separately. Face-to-face, semi-structured interviews were conducted by the researcher and the assistants, who have experience in conducting qualitative interviews. The researcher sought permission from the participants and audio-recorded the interviews. Although parent-adolescent dyads were recruited, interviews were held separately to avoid the potential of power dynamics to affect the quality of data obtained from the adolescents. Parents were not involved and were not present when adolescents were interviewed. The interviews were held in the presence of the researcher, and his assistants and the participant only.

The interviews lasted for 35 to 60 min. Data collection for the second phase of the study took place between August 2022 and January 2023.

*Data Analysis and Synthesis* in the qualitative study, data was analysed inductively following Braun and Clake’s approach to thematic analysis [26]. The doctoral student listened to the recorded interviews to familiarise himself with the data before and after transcription. Data was transcribed verbatim in Asante Twi or English. The interviews which were conducted in Asante Twi were translated into English after transcription before being retranslated into Asante Twi and confirmed, to ensure that participants’ voices were duly represented. After this, the doctoral student compared the transcript with the recorded interviews to check for accuracy with transcription. While participant verification of the English translations was not feasible in this instance, the multi-step translation and verification process was designed to uphold the integrity and accuracy of the data. The transcribed data was then uploaded into Atlas.ti software, version 23.0.7, for it to be organized into meaningful units to generate initial codes. The codes were put together to generate themes and subthemes, and these were refined to avoid overlap between the themes. Conclusions were drawn from the identified categories and themes to align with the objectives. The themes were defined and named, and then the report was produced.

### Mixed method integration

The aim of the mixed method integration was to describe how the qualitative findings explained the SR quantitative findings. The independent intramethod approach, where separate analysis is done for the quantitative and qualitative studies, was employed in the mixed analysis. Inferences were drawn from each analysis and the findings were compared for interpretations to be made.

A mixed data collection inventory was first taken which included as many data points as possible. Interpretive analysis was then done to interpret the data patterns. Back-and-forth exchange was used to identify data linkages. Joint displays were then used to organize linked data. The findings were examined for complementarity and divergence. The authors returned to theory to find explanations and examined intramethod findings for possible biases to handle divergence. Meta-inferences were then generated.

## Results

### Summary of quantitative results

Following a thorough search of databases, 1,706 studies were retrieved. Thirteen studies were included, following title and abstract screening. Five studies were included when full text screening and narrative synthesis were done because of the heterogenous nature of the studies. Four studies were RCTs and one used quasi-experimental design. Two of the studies that were included were from Iran and one each from Tanzania, South Africa, and Uganda. On average, the studies focused on adolescents between 13 and 16 years of age. One of the studies delivered the intervention to both parents and their adolescents. In the other four studies, interventions were delivered only to parents. The method of intervention delivery included role plays, lectures, group discussions, posters, games, and take-home assignments. Intervention delivery was done by experts such as a SRH education and adolescent counsellor, a consultant midwifery student with certificate in sexual training of children and adolescents, teachers, and HIV peer educators. The various interventions were delivered in health centers, community, worksite, and school settings. Some of the studies had components other than SRH communication, such as normal sexual development and condom use behaviour. SRH communication was found to be influenced by SRH information, motivation, or attitudes towards SRH information communication, and SRH communication skills.

### Summary of qualitative findings

Ten parent-adolescent pairs were selected to participate in the study. Four of the parents were males and six were females based. Nine of the participants were married and the other was divorced. Two of them had no formal education, whilst the rest had received formal education, with one ending at the primary level and the rest at the tertiary level. Eight of the participants were Christians and two were Muslims.

*Five themes emerged from the study* in the first theme, SRH topics that were discussed by parents as well as the sources of information for the discussion were labelled as *SRH information communicated*. The second theme related to the elements that either encouraged or discouraged parents from discussing SRH issues with their adolescents. This was labelled *individual parent and adolescent factors*. The third theme was about the perception of the parents regarding support from significant others and the behaviour of the community towards SRH communication with adolescents. This was labelled *contextual factors influencing SRH information communication*. The fourth theme was on how parents share SRH information with their adolescents. This was labelled *SRH communication skill needs of parents*. The last theme was on how parents would want SRH intervention to be packaged to meet their needs which considers the method of delivery, experts to deliver and venue for delivery of intervention and this was labelled *Context specific Information Communication Intervention*.

In the second qualitative phase, 10 adolescents of the parents interviewed in the first qualitative phase were selected. Their ages ranged from 13 to 16 years, based on the Systematic Review participant population. There were six females and four males. Seven of the participants were at the Junior High School level and the other three were at the Senior High School level. Eight of the participants were Christians and two were Muslims.

Five themes emerged from the study. The adolescent and parent factors that influenced the SRH communication between adolescents and their parents were labelled *adolescent and parent concerns that influence SRH communication skills*. Adolescents’ perception of the support from significant others, and cultural norms that influence their SRH communication with their parents, were labelled *sociocultural issues influencing communication skills*. The process by which adolescents share SRH information with their parents was labelled *SRH information communication that influences communication skills*. The question of the skills that are needed by the adolescents for communicating SRH information with their parents was labelled *SRH information communication skill needs*. The last theme was on how the adolescents would want SRH intervention to be packaged to meet their needs which considers the method of delivery, experts to deliver and venue for delivery of intervention and this was labelled *Context specific Information Communication Intervention*.

### Mixed method findings

**Fig. 2:**
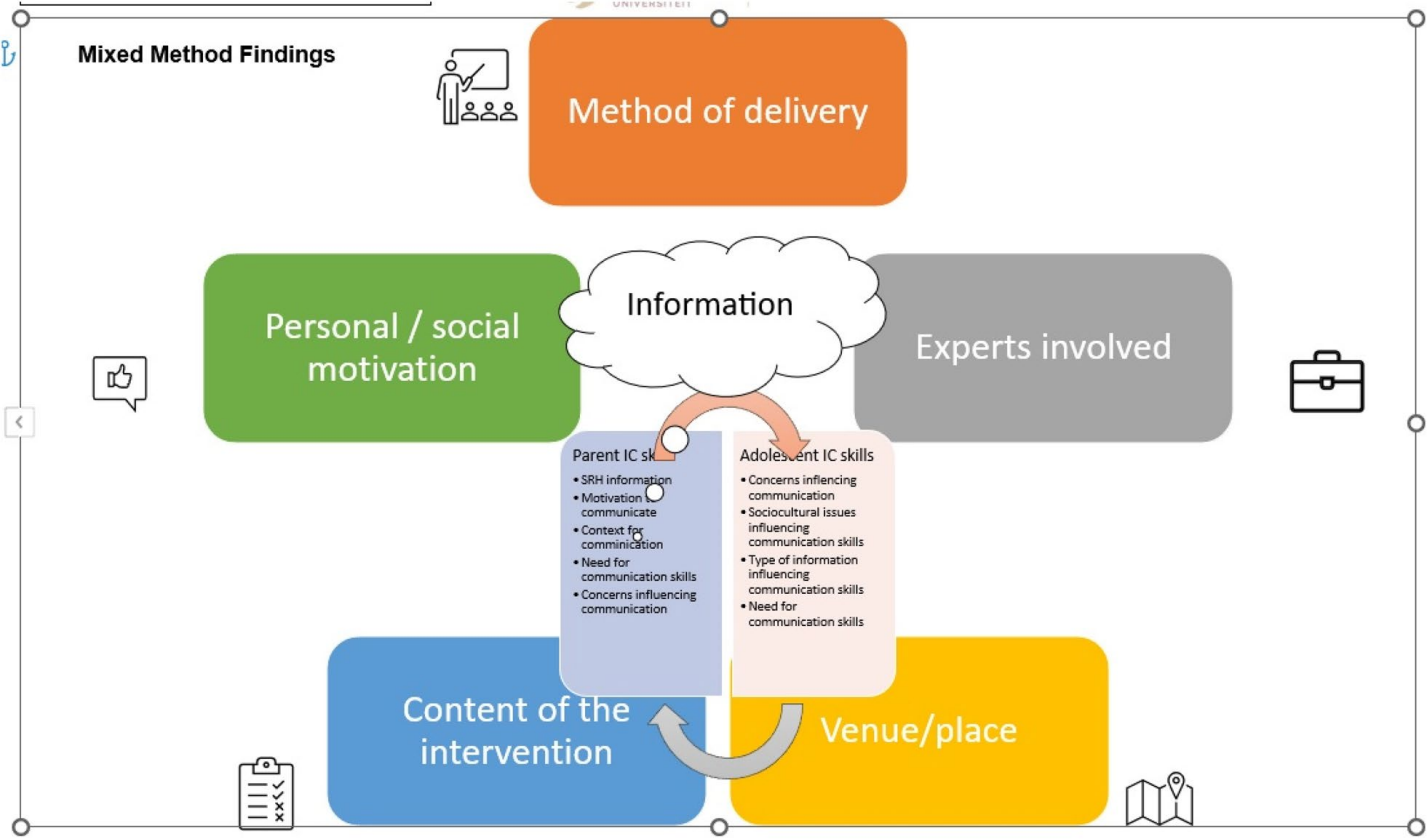
Mixed Methods Results

The findings from the Systematic Review and qualitative study revealed seven areas of focus which are, method of intervention delivery, experts involved in the delivery of intervention, venue or place of intervention delivery, SRH information, motivation, SRH information skills and SRH information communication (Fig. 2).

Regarding the method of delivery, the qualitative findings explained the quantitative findings in the Systematic Review in that, what emerged in the qualitative data were methods that had been used in the studies identified in the systematic review. Various methods were used across the identified interventions in the delivery. The methods included lectures, games, group discussions, role-plays and brainstorming [1]; workshop, classroom teaching (which included role plays, debates and writing exercises), homework assignments [27]; lectures, posters, group discussions, exercises, role-plays and creating scenarios [13]; workshop making use of group sessions [15]; group Counseling [9]. Parents in the qualitative study mentioned that they would prefer workshop with lectures, role plays, group discussions, role plays, discussion and brainstorming (Table 1). Adolescents preferred classroom teaching with homework and games (Table 1). The illustrative quotes have been given on Table 1. It is likely that the delivery method employed by Seif and colleagues [1] would be accepted in the Ghanaian context.

**Table 1:** Joint Display of Method of Delivery

Domain	Quantitative results	Qualitative findings (Parents and Adolescents)	Illustrative quotes	Meta-inferences
Method of Delivery	- Workshop, Classroom teaching (which included role plays, debates and writing exercises), Homework assignments [27] - Lectures, posters, group discussions, exercises, role-plays and creating scenarios [13] - Workshop making use of group sessions [15] - Group Counseling [9] - Lectures, games, group discussions, role-plays and brainstorming [1]	- Parents preferred a workshop with lectures, role plays, group discussions, role plays, discussion and brainstorming - Adolescents preferred classroom teaching with homework, games	“*In case there is something like that I will prefer that we do that through workshops,..they can give us lectures on that, or maybe group discussions or role-plays…something, at a point, we can discuss or brainstorm*.” (P7, M, 40 years) “*mmm…we can be taught in our classrooms then we are given a homework so that we can do it with our parents*” (A1, M, 16 years)	- Qualitative findings explain the quantitative findings - Different methods were employed in different studies in the Systematic Review to ensure cultural appropriateness - In this case, it is likely that, the methods mentioned by parents and adolescents in the qualitative methods may be accepted in the Ghanaian context

This Table 1 refers to the joint display of the method of intervention delivery as found in the systematic review and what will be preferred during the intervention delivery as they emerged from the qualitative study. What participants mentioned most in the qualitative study, including workshops and classroom teaching together with homework, fell within the various methods of delivery that were identified from the systematic review

Regarding the experts used to deliver the interventions (Table 2), the qualitative findings explained the quantitative findings in the Systematic Review in that, what emerged in the qualitative data were experts with similar background that had been used in the studies identified in the systematic review. Teachers [14]; expert in sexuality health education, adolescent counsellor [13]; peer HIV educators, clinical psychologists [15]; and consultant midwifery student [9] were found in the systematic review (Table 2). In the qualitative study, nurses and midwives also emerged as experts that would be preferred (Table 2). Parents only wanted that their children to be taught by someone who holds their culture in high esteem so that adolescents would receive a culturally appropriate SRH information.

**Table 2:** Joint Display of Experts delivering Intervention

*Domain*	Quantitative results	Qualitative findings (Parents and Adolescents)	Illustrative quotes	Meta-inferences
*Experts delivering Intervention*	- Teachers [14] - Expert in sexuality health education, Adolescent Counselor [13] - Peer HIV educators, Clinical Psychologists [15] - Consultant Midwifery Student [9]	Parents mentioned Nurses or Midwives and wanted their adolescents to be taught by these people, however they wanted these people to be religiously inclined so that they do not give any information that is not culturally sensitive Adolescents mentioned nurses as experts	“*I think this one, because it is health, the health professionals like nurses or midwives can do it, but they should be Christians so that they don’t spoil the moral values we have given to our children…better still we can be trained, and we will know how to talk to our adolescents and the kind of information to give to them*”. (P5, F, 39 years) “*The nurses can teach us this, they have been taking care of our health*” (A5, F, 15 years)	The qualitative findings explained the quantitative findings. In the qualitative findings, nurses and midwives were preferred because they are healthcare providers. However, parents also wanted someone who will take into consideration their cultural values when teaching their adolescents

This Table 2 covers the experts that were used to deliver interventions identified in the systematic review and what would be considered in the Ghanaian context. The parents expect the experts to deliver the intervention both to them and their adolescents which explains the findings from the systematic review. In this case, for it to be more culturally appropriate, parents expected that the expert should be a nurse or midwife who is religiously inclined so that their religious values will be maintained

In respect of the setting for the study (Table 3), the qualitative findings explained the findings in the systematic review. In the systematic review, school setting [14]; worksite [15]; community [1, 13]; health centre [9]. In the qualitative study, it emerged that parents mentioned that they would want to receive the training at a community centre. Adolescents preferred their school environment. Illustrative quotes have been given on Table 3.

**Table 3:** Joint Display of Setting for Intervention Delivery

*Domain*	Quantitative results	Qualitative findings (Parents and Adolescents)	Illustrative quotes	Meta-inferences
*Setting for Intervention Delivery*	- School setting [14], - Worksite [15], - Community [1, 13] - Health centre [9]	Parents mentioned that they would want to receive the training at a community centre Adolescents preferred their school environment	**“***We can be gathered in a community centre, mostly that is where most meetings are held…our adolescents can also have theirs in school or the same place…we just want to be sure of what they are going to tell them*” (P5, F, 39 years) “*It can be done in school; they can group us or maybe a very big classroom can be used*”. (A6, M, 16 years)	- The qualitative findings explain the findings from the systematic review - Community- or school-based intervention may be acceptable for parents and adolescents in the Ghanaian context - Parents, however, would prefer that adolescents have theirs at the community or the school

This Table 3 shows the setting for intervention delivery. Most parents preferred a community setting whilst the adolescents preferred it to be delivered in schools. This explained the findings from the systematic review. Parents cared about what information adolescents would be given, regardless of the setting, for the intervention delivery

With respect to the SRH information, only two studies [1, 15] measured the SRH information communicated by parents and adolescents. Various SRH information had been communicated by parents and adolescents as identified across the studies. These included the range of SRH topics. However, it was noted that in some studies, few topics were in discussed, why in others, most topics had been discussed. No single study had discussed all SRH topics. This has been well presented in article 1. In the qualitative study, parents and adolescents had discussed about pregnancy, STIs, pregnancy and STIs prevention, abortion, abstinence, sex, changes in adolescence, personal hygiene. These had been discussed across parents and adolescents. Contraceptives was not really discussed among majority of parents and adolescents. Most parents mentioned that they will not talk about it because of the age of the adolescents and others because it is not culturally appropriate. Information sources for parents included relatives, books, church, mosque, the media, personal experiences whilst parents, books, school, relatives, peers, church, mosque and the media. Illustration quotes have been given on Table 4.

**Table 4:** Joint Display of SRH Information Communicated

	Quantitative results	Statistical measures		Qualitative findings	Illustrative quotes	Meta-inferences
*SRH Information*	The scope of SRH topics communicated by parents and adolescents increased after the intervention delivery [b(SE) = 3.26(1.12), p = .005 [15]; but no significant difference was observed in Seif and colleague [1] These two studies measured SRH information as an outcome. However, all other studies reported on SRH information that was discussed during the intervention delivery	The parent / adolescent sexual communication scale [23] Likert scale Questionnaire	SRH topics discussed by parents and adolescents (pregnancy, STIs, pregnancy and STIs prevention, abortion, abstinence, sex, changes in adolescence, personal hygiene)		*"The focus was on the changes in the adolescent, the hormonal changes, the physical changes and attitudinal changes…the need for her to keep herself hygienically during menstruation" (P8, M, 48 years)* *“We discuss about teenage pregnancy, abortion and sexual transmitted disease and dating” (A9, F, 13 years)* *"We watch some on television, we use the internet to google and we have books that we read to find information about all these things." (P5, F,39 years)* *“TV (television), radio, friends, teachers and members in the community, I also hear that from my parents” (A8, F, 14 years)*	Qualitative findings explain the quantitative findings that not all SRH topics are communicated by parents, but interventions can promote an increase in SRH communication in general and increase the SRH topics discussed by parents and adolescents The sources of information from the qualitative studies explain the various forms by which interventions can be packaged to meet the communication needs of parents and adolescents

This Table 4 illustrates the SRH information that was communicated during intervention delivery in the systematic review and what parents and adolescents have communicated from the qualitative data. Various SRH topics were communicated in the delivery of the various interventions identified in the systematic review. After intervention delivery, some parents communicated few new topics they had not discussed with their adolescents before. The qualitative findings explained the quantitative data in that because of cultural values, topics that were culturally appropriate had been discussed between parents and their adolescents

On account of what motivated parents and adolescents to communicate, the qualitative findings explained the quantitative findings. This was measured by Seif and colleagues [1] and [14] in their studies. Personal and social motivation emerged in the qualitative study to be factors that influence SRH information communication skills and the search for SRH information Table 5).

**Table 5:** Joint Display of Motivation to Communicate SRH Information

*Domain*	Quantitative results	Statistical measures	Qualitative findings (Parents and Adolescents)	Illustrative quotes	Meta-inferences
*Motivation to Communicate SRH Information*	Parents and or adolescents were motivated personally to communicate SRH information after the intervention delivery [effect size (d = 0.3) [1] [0.20 (t = 2.772; p = 0.006) for parents and adolescents respectively [14]	Likert scale Questionnaire	• Personal motivation • Social motivation	*"It is very important because if you don’t share the right information they may be misinformed. They may get it from the wrong source and, in the end, it will not help them. So, they need to get it from somebody who has knowledge and can guide them." (P9, M, 63 years)*	The qualitative results explain the findings of the systematic reviews that parents and adolescents have a positive attitude towards SRH information communication in general. It is some topics that they are rather not motivated personally to communicate
*Motivation to Communicate SRH Information*	Social norms did not change significantly after the intervention delivery in the interventions that measured social norms. [F (1, 827) = 0.46, (p = 0.51). [1]	Likert scale Questionnaire	Personal motivation Social motivation	“*I know my parents can give me the best and appropriate advice and what I have to do is to talk to them about it*”. (A2, F, 13 years) *"In our culture, there are some parts of the human body, sexual parts, you can’t mention them. You must speak about it proverbially. If you speak too straight about it, you are seen to be a bad person." (P8, M, 48 years)* “*Sex is not something that they talk about in this community, but I don’t also know why”. (A7, M, 13 years)*	The qualitative findings explained the quantitative findings that before intervention delivery and even after intervention (to some extent) social norms have influence on SRH communication. That is why after the intervention delivery, social norms did not change significantly

This Table 5: The qualitative data explained the motivation for SRH communication between parents and adolescents in the systematic review. There were parent and adolescent factors that either acted as a barrier or facilitator to SRH information communication. Also, the cultural norms either facilitated or hindered communication on certain SRH topics. This explains why some topics were not discussed by some parents and their adolescents in some of the interventions. It also explains why social norms did not change significantly after intervention delivery, in the studies that measured social norms

Regarding the SRH information communication skills, the qualitative findings explained the quantitative findings. Those who were trained in the various interventions identified in the systematic review had improved SRH information communication skills as compared to those in the control group. In the qualitative study, because no intervention had been delivered, it was found that parents and adolescents lacked SRH information communication skills (Table 6). This explains the need for a culturally sensitive SRH information communication intervention to be used to train parents and adolescents on SRH information communication.

**Table 6:** Joint Display of SRH Information Communication Skills

*Domain*	Quantitative results	Statistical measures	Qualitative findings	Illustrative quotes	Meta-inferences
*Need for communication skills*	There was an increase in self efficacy and actual ability to communicate after the intervention delivery [F(1, 827) = 10.81, (p ≤ .001) [1]] [(M = 5.0, SD = 1.7)	Likert scale Questionnaire	- Actual ability to communicate - Nature of SRH information communication	*"We are free, we talk, we laugh, so it is normal conversation. It is not something strict, I don’t sit her down strictly, no no no, we just communicate as we have been doing always."* (*P5, F, 39 years*) *“It is difficult for me. Because I have not heard about those things before. I don’t do those things (sex) that is why it is difficult for me, and I don’t really like talking about those things (sex).” (A7, M, 13 years)*	The qualitative findings explain the state of parents and adolescents before intervention delivery in the various studies identified in the systematic review. They were not skilled enough to communicate but had the ability to communicate on some topics such as menstruation
	[12] [(p < 0.001) [13]			*"I have warned you, don’t be walking with women; I’ve told you that this boy I don’t like him coming to my house with you…" (P9, M, 63 years)* *“Sometimes when my mother talks, I don’t understand…she normally adds proverbs. She normally will say that when I grow, I will understand.” (A6, M, 16 years)*	However, after the interventions, they had improved skills to communicate SRH information on some additional topics

This Table 6: This displays the SRH IC skills that is needed to communicate SRH information. Data gathered from the qualitative study explained the quantitative study. There were identified skill needs in the qualitative study. This may be bridged when parents are trained to get skills as shown in the systematic reviews

Lastly SRH information communication was also identified as a theme (Table 7). Regarding this, the qualitative findings explained the quantitative findings from the systematic review. After the various interventions, frequency of communication improved. In the qualitative study, it emerged that parents and adolescents do not frequently communicate SRH information communication. It is believed that, when there is an intervention, it could assist in training them to have the skills that will translate into frequent SRH information communication.

**Table 7:** Joint Display of SRH Information Communication

*Domain*	Quantitative results	Statistical measures	Qualitative findings (Parents and Adolescents)	Illustrative quotes	Meta-inferences
*SRH Information Communication*	Parents and adolescents were able to communicate with openness and comfort and could communicate frequently, unlike before the delivery of the various interventions ([p value < 0.001 [6]; [bSE = .98(.39), p = .02 [12]) [F(1, 827) = 16.74; (p ≤ 0.01) with small effect size (d = 0.3) [1]]; (p < 0.4, [10])	Likert scale Questionnaire	SRH Information communication	*"We were lying down on the bed, comfortably lying down on the bed. It was like a conversation." (P10, F, 50 years)* *“We talk about it once a while” (A9, F, 13 years)* *"To be honest it isn’t frequent, I have done it twice." (P7, M, 40 years)* *“I felt uneasy, I felt anxious…this is because I have not been talked to about such matters before” (A6, M, 16 years)*	The qualitative findings explain the quantitative findings from the systematic review that before the intervention delivery parents were not open to their adolescents, could not communicate comfortably and frequently and adolescents were the same

This Table 7 Data gathered from the qualitative studies explain that of the systematic review. Some parents and adolescents had communicated on some SRH topics but infrequently and with various sentiments. After intervention delivery in the various studies identified in the systematic review, there was improved communication, even though not sustained in all the interventions. This is promising and shows that when the contextual factors are taken into consideration, an adapted intervention from the ones identified may be able to train parents and adolescents to communicate effectively regarding SRH information

## Summary of mixed method findings

It was identified that the success of SRH information communication is determined by the skill possessed by the parents and their adolescents. For the communication to take place successfully, information that is culturally appropriate, which considers the cultural values as a motivation is likely to improve the attitudes of parents and adolescents towards SRH information communication.

A culturally sensitive SRH information communication intervention must consider the method of delivery, the place of delivery and the experts involved in the delivery. Making use of classroom teaching in a school for adolescents, and workshops in the community for parents, may be culturally appropriate and explains some of the methods that were used in the various interventions identified in the quantitative phase. Health professional, especially nurses and midwives, who value the cultural norms may be contextually appropriate, which explains the findings from the quantitative phase. Parents would want to know what their adolescents are being taught and would prefer that they are present.

## Discussion

This study was to systematically integrate data from a systematic review of effective SRH information communication interventions in LMICs and a qualitative study in Ghana. This was done to know the components to consider when adapting a SRH information communication intervention into the Ghanaian setting. The study highlights important components of SRH information communication interventions, and the culturally sensitive components that can be factored into them, in order to have a contextually appropriate intervention for Ghanaian parents and adolescents. This is necessary because interventions are more likely to be accepted if they have values, beliefs and behavioural sensitivity that meet those of the user [28].

Some barriers, such as social norms and lack of skilled health professionals, have been identified as hinderances to the acceptability of SRH interventions [29]. This being the case, paying attention to the voice of the participants and their preferences will help to adapt an intervention so that it is culturally sensitive and likely to be accepted by parents and adolescents in the Ghanaian context. Healthcare professionals such as nurses and midwives are likely to deliver the interventions effectively. This finding in the mixed method integration has been implemented in many SRH interventions [29]. Some interventions have also used a multidisciplinary health team for delivery [30]. What is generally known is that intervention studies that involve adolescents make use of those who directly care for adolescents or experts in adolescent health [32]. Thus, healthcare workers who have been trained to specially care for adolescents, especially with regards to SRH, will be beneficial and contextually appropriate for the intervention delivery. The concern of parents was that the healthcare professionals delivering the intervention should be familiar with their cultural values so that they are not compromised.

Another relevant finding of the mixed method integration concerns the setting for intervention delivery. This is the venue where the intervention delivery takes place. It emerged that a school-based setting or a community setting for adolescents and a community setting for parents are preferred. Most interventions in SRH for adolescents have used school-based settings, making use of a group format [33], and this has been effective. For instance, Barron [34] and Punamki and colleagues [35] used school-based settings with a group format and it was effective. Adolescents may feel comfortable in schools because they may be used to the environment and might have already have been having group discussions there. Using what they are interested in may lead to SRH information communication skill acquisition to communicate SRH information. A community-based approach for parents and or adolescents has been effective in some interventions [36, 37].

Regarding the method of delivery, it was found that parents and adolescents preferred role play, lectures, group discussions and teaching which were also used in the various interventions that were identified in the systematic review. Different delivery modalities, such as lectures, have been employed in various studies to deliver SRH programmes for adolescents, such as in China where there is conservativeness in adolescent SRH [38]. There is the possibility of these methods working effectively in a SRH information communication intervention delivery since they have been used in similar interventions in LMICs, as identified in the systematic review.

SRH information that is usually communicated by parents and adolescents is influenced by social norms [1]. In the Ghanaian context, as identified from the qualitative data, the SRH information that has been mostly communicated by parents and their adolescents has included menstruation, STIs, pregnancy, abstinence, changes in adolescence, sex and sexual relationships. This supports earlier studies in Ghana [39, 40]. This explained the data gathered from the systematic review in phase one. SRH is a sensitive area, especially regarding adolescents, and parents always want to preserve their cultural values. For instance, in the implementation of the DARAJA curriculum in Zanzibar, parents opposed the discussion regarding condom use and other contraceptive methods with adolescents, and consequently that part was omitted before the implementation [1].

In South Africa, while stigma persists, interventions targeting parents to facilitate open discussions have shown promise, demonstrating that culturally sensitive approaches can mitigate these barriers [41]. Studies in India, such as those by Mehra and colleagues [42], show comparable challenges, with family members avoiding conversations about contraception and sexual health due to traditional beliefs about purity and morality.

This suggests that what may be accepted in the Ghanaian context is what has been mentioned by participants in the qualitative data from Ghana. Therefore, this must be taken into consideration in the adaptation of the SRH information communication for parents and adolescents in Ghana. This is because the effectiveness of interventions in public health is usually associated with the social and cultural context in which the implementation takes place. An effective intervention may not be effective in a new setting [43].

The varying effectiveness of SRH communication interventions across contexts can be attributed to a range of contextual factors. Cultural norms play a pivotal role; in settings where SRH discussions are culturally taboo, interventions often face resistance, as seen in regions with conservative social norms [44, 45]. Conversely, interventions in culturally open societies tend to achieve greater success.

Caregiver comfort levels are another critical factor. Interventions that include caregiver training to build confidence and communication skills are more effective. However, caregivers who feel unprepared or perceive SRH discussions as promoting inappropriate behaviors may limit the success of such interventions [45].

Adolescent receptiveness also significantly affects outcomes. Adolescents who view their caregivers as approachable and nonjudgmental are more likely to engage in meaningful conversations. Interventions in such contexts have shown higher effectiveness. In contrast, authoritarian or dismissive parental attitudes can reduce intervention impact, highlighting the importance of relational dynamics in SRH communication [46].

The findings also suggest that parents and adolescents need skills to communicate SRH information. This supports an earlier study in Ghana [47]. Lack of required skill can be a barrier to SRH information communication [40]. This highlights the need for intervention to train parents and adolescents to develop skills for SRH information communication. Appropriate delivery methods are believed to contribute to SRH information communication skill development [1]. Evidence from the literature posits that the openness and frequency of SRH information communication may increase when parents and adolescents possess the skill for communicating such information [48].

## Strength and limitations

The qualitative phase provided data that is contextually appropriate because this will inform the adaptation of a culturally sensitive SRH information communication intervention. The integration of results from the systematic review of SRH information communication interventions and the qualitative data which made use of rigorous methods increases the value of the findings.

Only studies reported in English were considered in the systematic review; studies in other languages were therefore excluded. The age of adolescents was limited to 13 to 16 years, and this excludes the needs of adolescents and parents of adolescents and the interventions where the mean age was lower or higher than the stated age range.

## Conclusion

This study used an explanatory sequential mixed method approach to systematically integrate data from a systematic review of effective SRH information communication interventions in LMICs and a qualitative study in Ghana. The qualitative data explained the quantitative data from the systematic reviews and has provided the basis for the adaptation of a culturally sensitive SRH information communication intervention in Ghana. The study reports on the methods of delivery, the use of experts in delivery and the delivery settings which are culturally appropriate. In all, parents and adolescents have the desire to communicate SRH information. However, they want to share information that preserves their cultural values. Both parents and adolescents lacked skills to share this information. So SRH information communication intervention which is culturally sensitive may help to improve communication skills for parents and adolescents.

The development of localized SRH education curricula that involve community stakeholders, including parents, traditional leaders, and educators are therefore recommended. These curricula would emphasize culturally appropriate ways to discuss sensitive topics with adolescents. There should be formulation of policies that institutionalize parent-focused SRH workshops as part of community health initiatives. Such workshops can equip parents with the skills and confidence to navigate SRH discussions within their cultural contexts.

## Data Availability

No datasets were generated or analysed during the current study.
